# Effect of salt mixture on flavor of reduced‐sodium restructured bacon with ultrasound treatment

**DOI:** 10.1002/fsn3.1679

**Published:** 2020-05-29

**Authors:** Yu Zhou, Yu Wang, Qiong Pan, Xi‐xi Wang, Pei‐jun Li, Ke‐zhou Cai, Cong‐gui Chen

**Affiliations:** ^1^ School of Food and Biological Engineering Hefei University of Technology Hefei China; ^2^ Engineering Research Center of Bio‐process from Ministry of Education Hefei University of Technology Hefei China; ^3^ Key Laboratory on Deep Processing of Agricultural Products for Anhui Province Hefei China

**Keywords:** lipid and protein oxidation, reduced‐sodium restructured bacon, salt mixture, ultrasound, volatile flavor compound

## Abstract

Flavor loss from sodium reduction is a large challenge faced in the meat industry. The effects of salt mixture (KCl: CaCl_2_ = 1:1, w/w) content (0%–1.0%) on flavor of reduced‐sodium (1.5% NaCl) restructured bacon with ultrasound treatment (UT, 600 w for 30 min) were investigated. The results showed that 0.5% salt mixture (0.25% KCl and 0.25% CaCl_2_) could significantly (*p* < .05) enhance the lipid oxidation, the protein oxidation, and the formation of free amino acids of reduced‐sodium UT‐restructured bacon and could also markedly (*p* < .05) improve its flavor and the overall quality of sensory evaluation via promoting the release of five kinds of volatile phenolic compounds (o‐cresol, m‐cresol, 2‐methoxy‐phenol, 2‐methoxy‐4‐methylphenol, and 2‐methoxy‐5‐methylphenol) and the formation of five kinds of volatile aldehyde compounds (hexanal, nonanal, decanal, furfural, and 5‐methyl furfural). It is interesting to understand the mechanism for the effect of salt mixture on flavor and to efficiently develop a technique for improving the flavor of reduced‐sodium products in the meat industry.

## Graphical abstract

Salt mixture promoted lipid and protein oxidation of the reduced‐sodium UT‐bacon. The 0.5% mixture promoted the release and formation of key volatile flavor compounds. It is promising to develop a technique for improving the flavor of low‐salt bacon.

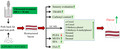

## 1 INTRODUCTION

It is well known that sodium chloride (NaCl) can improve processing characteristics, ensure food safety, enhance the product texture, and contribute to food flavor (Wen et al., 2019). However, a high intake of sodium salt is associated with high health risks, including cardiovascular diseases, stroke, and hypertension (Kloss, Meyer, Graeve, & Vetter, 2015). The World Health Organization convened an assembly to set a global target that salt intake should be reduced by 30% by 2025 and recommended that the salt intake of an adult should be <5 g/day (<2 g sodium/day) (WHO, 2012). Thus, the food industry should produce more low‐sodium foods to reduce the risk of sodium‐related diseases.

Meat products, such as bacons and hams, contribute 15%–25% of the salt/sodium in a person's diet (Kloss et al., 2015). Moreover, bacon is an important share of the processed meat products. An adult consumes approximately 0.19 g/kg body weight/day of bacon in Europe (EFSA, 2015). It is reported that the declared salt content of bacon is between 2.39% and 3.19%, and the salt levels are higher than in ham products in selected European countries (Delgado‐Pando et al., 2018). In recent years, restructured bacon has also developed rapidly and has become an important part of meat products in the Chinese market. Therefore, there is a lot of room to reduce sodium salt in restructured bacon in order to ensure the healthy diet of consumers.

The quality, such as flavor, color, and appearance, is a dominant factor to determine the meat character and purchasing decision of the consumers. However, directly decreasing the salt content in meat products can lead to flavor loss, texture deterioration, shelf life shortening, more rapid growth in natural flora (Desmond, 2006; Jeremiah, Ball, Uttaro, & Gibson, 1996), and reduced consumer purchasing intentions (Delgado‐Pando et al., 2018). Salt reduction also affects the development of those volatile compounds (Corral, Salvador, & Flores, 2013). Accordingly, determining how to enhance the flavor of reduced‐sodium restructured bacon is a significant challenge.

Ultrasound treatment can significantly increase the relative contents of volatile flavor compounds, especially aldehydes, ketones, and alcohols, and then produce a better flavor in spiced beef (Zou et al., 2018), especially improve the taste and purchase intention of ham with 0.75% of salt (Barretto, Pollonio, Telis‐Romero, & da Silva Barretto, 2018). Moreover, the addition of KCl and CaCl_2_ into low‐sodium meat products can also improve the flavor and overall acceptance (de Almeida, Villanueva, da Silva Pinto, Saldaña, & Contreras‐Castillo, 2016), increase the degree of lipid oxidation (dos Santos, Campagnol, Fagundes, Wagner, & Pollonio, 2017), and promote the content of amino acids (dos Santos et al., 2015). And the best sensory acceptance can be yielded in mortadella when 50% NaCl was replaced by 25% CaCl_2_ and 25% KCl (Horita, Morgano, Celeghini, & Pollonio, 2011), although the replacement of NaCl by 50% of KCl or CaCl_2_ could lead to bitter taste of dry‐cured ham (Armenteros, Aristoy, Barat, & Toldrá, 2012) or texture deterioration of salted meat (Vidal, Bernardinelli, Paglarini, Sabadini, & Pollonio, 2019). CaCl_2_ has also a greater lipid oxidation capacity when compared to NaCl and KCl (Nachtigall et al., 2019) and is not well accepted. The mixture of KCl and CaCl_2_ may be an interesting strategy depending on the meat matrices. To our knowledge, no literature has reported the effects of both KCl and CaCl_2_ on oxidation and flavor compounds of reduced‐sodium restructured bacon with ultrasound treatment.

The objective of this work was to investigate the effect of the salt mixture (KCl: CaCl_2_ = 1:1, w/w) at different content levels (0%–1.0%) on the flavor compounds of reduced‐sodium restructured bacon (1.5% NaCl) with ultrasound treatment (UT, 600 w; 30 min).

## 2 MATERIALS AND METHODS

### 2.1 Materials

Fresh pork back fat and lean pork of *Landrace* pig were purchased from the Hejiafu Supermarket (Hefei City, Anhui, China) and stored at 4°C until use (within 4 hr). The pigs were slaughtered in compliance with ethical guidelines for animal care published by the Ministry of Science and Technology of the People's Republic of China in 1988. The composition of the lean pork was 71.8% water, 18.2% crude protein, and 5.6% crude fat. The composition of the back fat was 6.1% water, 2.1% crude protein, and 91.3% crude fat (AOAC, 1996). Food‐grade NaCl, KCl, and CaCl_2_ were purchased from Yinuo Biotechnology Co., Ltd. Food‐grade sodium nitrite, soy protein isolate, sodium tripolyphosphate, and sugar were purchased from Jinshan Pharmaceutical Co., Ltd. Liquid smoke was purchased from Jinan Hualu Food Co., Ltd. Commercial bacon (Smithfield, the smoked wood used in the bacon was the same as the raw material of the liquid smoke) was purchased from Shineway Group. Other reagents and chemicals in the research were of analytical grate or better.

### 2.2 Preparation of the reduced‐sodium restructured bacon

The ratio of fat to muscle (3:7, w/w) of the restructured bacon was determined via investigating the fat ratio of bacon in the Chinese market. The salt mixture contained KCl and CaCl_2_ in a ratio of 1:1 (w/w), according to the result of Horita et al. (2011). The pork back fat and lean pork were minced through a meat grinder (MG‐1220, Shunde Jinyimei Electrical Appliances Co., Ltd.). The salts, liquid smoke, and the other ingredients were sequentially added to the minced meat as depicted in Table 1 and were thoroughly mixed in a mixer machine (CL‐877, Chulaikesi Electrical Equipment Co., Ltd.) at 60 rpm for 5 min. The mixed meat was put into a polyethylene bag and sealed (approximately 200 g) by a vacuum‐packing machine (DZ‐260, Rayleigh Packaging Machinery Co., Ltd).

**TABLE 1:** Content of NaCl and salt mixture in reduced‐sodium restructured bacon under ultrasound treatments (U0, U1, U2, and U3)^a^

Treatments of four	Amount of salts added in g/100 g meat		
	NaCl	KCl	CaCl_2_
U0 (1.5%NaCl + UT 600 w) (Control)	1.5	0	0
U1 (1.5%NaCl + 0.125%KCl + 0.125%CaCl_2_ + UT 600 w)	1.5	0.125	0.125
U2 (1.5%NaCl + 0.25%KCl + 0.25%CaCl_2_ + UT 600 w)	1.5	0.25	0.25
U3 (1.5%NaCl + 0.50%KCl + 0.50%CaCl_2_ + UT 600 w)	1.5	0.5	0.5

Note

The content of other additives in U0‐U3 were the same and were 0.5% sugar, 0.01% sodium nitrite, 2% soy protein isolate, 0.4% sodium tripolyphosphate, and 0.5% liquid smoke, 11.1% water, respectively.

Abbreviation: UT, ultrasound treatment.

^a^ Deviation of weight of all salts was not in excess of ± 0.5%.

The sealed bag of mixed meat and a Ф15 probe were fully immersed in a 3,000‐mL beaker filled with ice water and were subjected to UT for 30 min under 600 w at a frequency of 20 kHz (Zou et al., 2018) using a SCIENTZ‐ⅡD ultrasound processor (Ningbo Xinzhi Biotechnology Co., Ltd.). The constant distance between the bag and probe head was 20 mm on the basis of the result of Kang et al. (2016).

The restructured bacon was prepared on the basis of the procedures described by Aaslyng, Vestergaard, and Koch (2014) and preliminary tests. The meat batter from cured bags, after UT and being stored at 0–4°C for 12 hr, was cooked at 72°C for 60 min in a stainless‐steel mold of bacon, followed by drying at 65°C for 60 min using an electric blast drying box (DGF30/7‐2, Nanjing experimental instrument factory), and then cooled. The restructured bacon was vacuum‐packed before slicing (2.8 mm thick) and frozen to −18°C. The bacon was thawed approximately 4 hr at 4°C before the values of thiobarbituric acid reactive substances (TBARS), protein carbonyl content, free fatty acids, and free amino acids were measured.

### 2.3 Determination of TBARS

Thiobarbituric acid reactive substances values of the restructured bacons were determined as described by Li et al. (2019). Five grams of the minced restructured bacon was mixed with 50 ml of a 7.5% aqueous solution of trichloroacetic acid (TCA) containing 0.01% ethylene diamine tetraacetic acid (EDTA) in a 150‐mL conical flask. These conical flasks were incubated at 50°C for 30 min in a thermostatic water bath oscillator (WE‐3, Ouyi Co., Ltd.) at 150 rpm. After filtering through double‐layer filter paper, 5.0 ml of filtrated volume was mixed with 5.0 ml of 0.02 M aqueous thiobarbituric acid (TBA) in a 25‐mL stoppered colorimetric cylinder. These cylinders were incubated at 90°C for 30 min in a thermostat water bath (HH‐56, Jintan Guosheng Experimental Instrument Factory) and were then cooled for 5 min in running water. Absorbance was measured at 532 nm using a spectrophotometer (UV‐6000PC, Yuanxi Instrument Co., Ltd.) against a blank containing 5 ml of 7.5% TCA and 5 ml of TBA reagent. The results, expressed as milligrams malondialdehyde (MDA)/kg meat, were calculated from the standard curve prepared by TEP (1,1,3,3‐tetraethyoxypropane). Each treatment was performed for three times, and the determinations were replicated three times.

### 2.4 Determination of protein carbonyl content

The protein carbonyl content was determined in accordance with the method described by Berardo et al. (2016). Carbonyl was reacted with 2,4‐dinitrophenyl hydrazine (DNPH) to produce the hydrazones of protein, which were detected by measuring the absorbance at 370 nm. The protein carbonyl content was calculated as nmol carbonyl/mg protein based on an absorption coefficient of 22,000 M^−1 ^cm^−1^. Each treatment was performed for three times, and the determinations were replicated three times.

### 2.5 Free fatty acid analysis

The free fatty acid (FFA) composition of the restructured bacon was analyzed as described by Huang, Li, Huang, Li, and Sun (2014). FFAs were converted to fatty acid methyl esters (FAMEs) and analyzed using a gas chromatograph (Agilent 7,693, Agilent Technologies Inc. US) equipped with a capillary column (DB‐WAX, 30 m length, 0.25 mm diameter, 0.25 μm film thickness; Agilent Technologies Inc. US). The injector and detector temperatures were both set at 250°C. The oven temperature program was 60°C for 1 min and then increased at 50°C/min up to 200°C, where it was maintained for 1 min, then increased to 250°C at 3°C/min and held at 250°C for 3 min. Helium was used as the carrier gas at a flow rate of 3 ml/min, and the split ratio was 10:1. Identification of the FAMEs was carried out by comparing their retention times with those obtained for standards. The results are expressed as the FFA percentages of the total FFAs. Each treatment was performed for three times, and the measurements were performed in triplicate.

### 2.6 Free amino acid analysis

The free amino acid (FAA) content in the restructured bacon was analyzed utilizing an Amino Acid Automatic Analyzer (L‐8900, Hitachi) and was determined according to the method described by Zou et al. (2018). One gram of the minced restructured bacon was placed in a centrifuge tube and brought to 10 ml with 5‐sulfosalicylic acid (40 g/L), after which it was shaken and mixed by vortex mixer (SA8, Mercy Scientific Instruments Co., Ltd.). After incubating it in a dark place for 1 hr, the tube was centrifuged at 10,000 *g* at 4°C for 30 min in a high‐speed freezing centrifuge (CT14RD, Tianmei Biochemical Instrument Engineering Limited Company). Finally, the supernatant was filtered through a 0.22‐μm membrane, and the resulting filtrate was analyzed by the automatic analyzer. Each treatment was performed for three times, and the measurements were performed in triplicate.

### 2.7 Analysis of volatile compounds

Volatile compounds of the restructured bacon were analyzed as described by Wen et al. (2019). The restructured bacon was baked in an oven at 210°C for 10 min and immediately minced, and five grams of the minced restructured bacon was packed into 20‐ml headspace sample vials (Supelco). The volatile compounds in the restructured bacon were collected in a solid phase microextraction fiber (50/30 μm DVB/CAR/PDMS, Supelco) and exposed to the headspace sample vials for absorption at 60°C for 40 min. After absorption, the fiber was immediately inserted into the GC syringe port and was then thermally desorbed at 250°C for 3 min. Volatile compounds were analyzed utilizing a GC‐MS system (QP2010 Shimadzu) with a DB‐5 ms capillary column (60 m length, 0.25 mm diameter, 0.25 μm film thickness; Agilent Technologies Inc.). Helium was used as the carrier gas at a flow rate of 1 ml/min, and the split ratio was splitless. After desorbing, the oven temperature was held at 40°C for 2 min, followed by an increase at 5°C/min to 60°C, with a subsequent increase to 100°C at 10°C/min and a final increase to 240°C at 18°C/min, where it was maintained for 6 min. The mass‐selective detector was applied 250°C as the ion source temperature. The ionization potential of the detector was 70 eV, and the mass spectrometer scan range was 33–350 m/z. The identification of volatile compounds was achieved by comparing their mass spectra with those in the NIST 11 library. The area of each peak was integrated and reported as an indicator of volatile compounds from the restructured bacon. Each treatment was performed for three times, and the measurements were performed in triplicate.

### 2.8 Determination of the key volatile compounds

The relative odor activity value (ROAV) was calculated to determine the key volatile compounds of the restructured bacon as described by Zhuang et al. (2016). The odor threshold values of those compounds were taken from the relevant literature (van Gemert, 2003; Giri, Osako, Okamoto, & Ohshima, 2010; Kosowska, Majcher, Jeleń, & Fortuna, 2018; Söllner & Schieberle, 2009).

### 2.9 Sensory analysis

Sensory evaluation of the restructured bacon was implemented by a nine‐member trained team according to the method described by Soladoye et al. (2017). The restructured bacon was baked in an oven at 210°C for 10 min and was then served on a white plate with two‐digit codes in a randomized order. A nine‐point scale was utilized, and the intensities of all sensations (aroma, taste, color, texture and aftertaste) were expressed from 1 (none of the sensation) to 9 (maximum of the sensation). A commercial bacon was used as an evaluation reference (7 points). And the overall quality was calculated using a weighted rubric (30% aroma, 30% taste, 10% color, 10% texture, and 20% aftertaste). During sensory evaluation, water at room temperature was provided to panelists for palate cleansing and to remove residual flavors between each sample.

### 2.10 Statistical analysis

Statistical analysis of the data was performed using IBN SPSS Statistics software (SPSS Inc.25) and was expressed as the mean ± standard deviation (*SD*). A one‐way analysis of variance (ANOVA) with Duncan's multiple range tests was used to determine the significance of the treatment effects (*p* < .05).

## 3 RESULTS AND DISCUSSION

### 3.1 Lipid oxidation and protein oxidation

**FIGURE 1:**
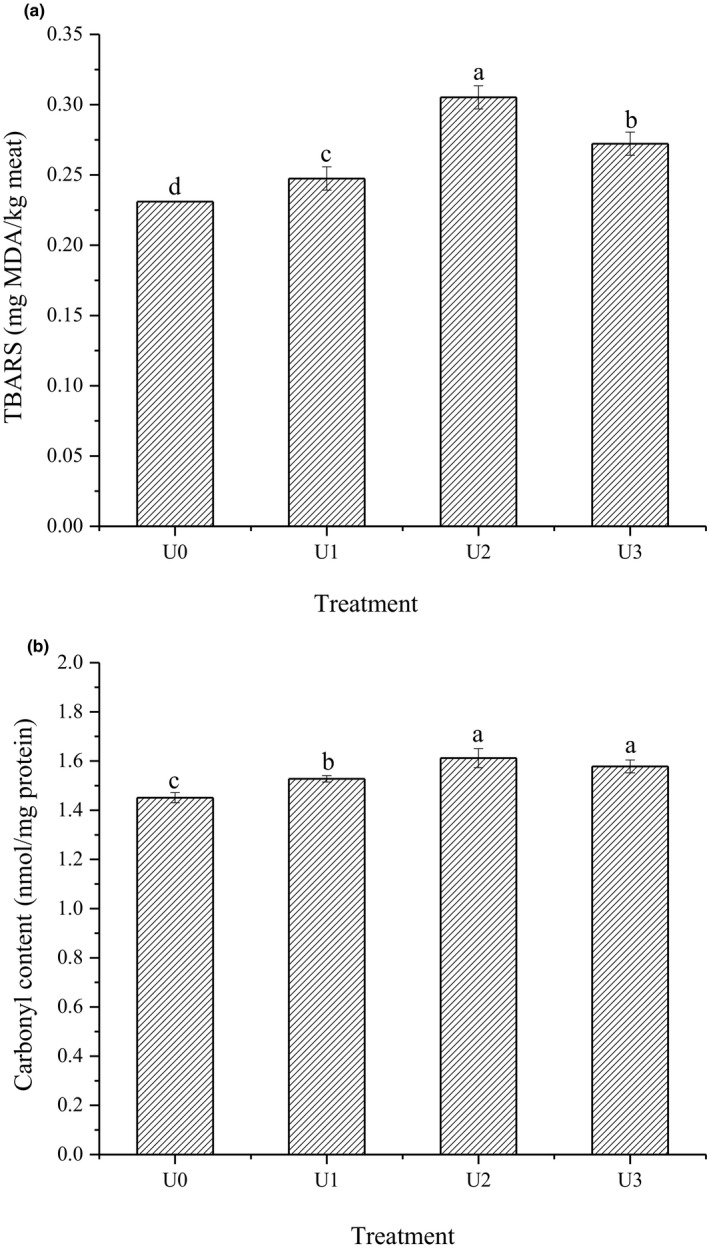
Lipid oxidation (a) and protein oxidation (b) of the reduced‐sodium restructured bacon under ultrasound treatment as affected by the content of salt mixture. ^a–d^ in the column indicates that the different letters are significantly different (*p* < .05, *n* = 3). *U0: 1.5%NaCl + UT 600 w; U1: 1.5%NaCl + 0.125%KCl + 0.125%CaCl_2_ + UT 600 w; U2: 1.5%NaCl + 0.25%KCl + 0.25%CaCl_2_ + UT 600 w; U3: 1.5%NaCl + 0.50%KCl + 0.50%CaCl_2_ + UT 600 w

Thiobarbituric acid reactive substances is a major index to evaluate the extent of lipid oxidation, which is primarily used to determine the secondary products of lipid oxidation (Wen et al., 2019). As shown in Figure 1a, the TBARS values of all of the treatment groups (U1, U2, and U3) were significantly higher than that in the control group (U0) (*p* < .05), with the maximum value being observed in U2. These results indicated that the mixture of both KCl and CaCl_2_ could promote lipid oxidation, and an intermediate level (0.5%) of salt mixture resulted in the highest prooxidative potential on lipids. It has been reported that replacing NaCl with KCl and CaCl_2_ could increase the lipid oxidation in sausages (dos Santos et al., 2017) and CaCl_2_ could induct more lipid oxidation (Nachtigall et al., 2019). KCl and CaCl_2_ may inhibit antioxidant enzyme activities in meats, thus facilitating lipid oxidation (Rhee, Smith, & Terrell, 1983). The TBARS contents usually increase with increasing ionic strength of the processed meats in a certain range (Hernández, Park, & Rhee, 2002). Additionally, the UT could generate free hydroxyl radical by cavitation and promoted the effective diffusivity of salt mixture and NaCl (Kang et al., 2016; Zou et al., 2018) and further promoted the oxidation of the restructured bacon by the salt mixture. The increasing trend of TBARS content from U0 to U2 could be attributed to the enhanced ionic strength (Table S3) that was induced by the addition of salt mixture and the diffusion of the salt mixture promoted by UT in the restructured bacon. However, high ionic strength can decrease the solubility of oxygen, exhibiting an inhibitory effect on lipid oxidation (Sharedeh, Gatellier, Astruc, & Daudin, 2015), thus resulting in the decreased TBARS value (*p* < .05) of the U3 group compared with the U2 group (Figure 1a). This result is similar to that described in some reports (Nachtigall et al., 2019; dos Santos et al., 2017). In addition, it should be noted that the lipid oxidation could not be the primary cause of the change in the bacon quality because of its low TBARS values.

Formation of carbonyl compounds is a major indicator of protein oxidation in meat products (Estévez, 2011). Carbonyl groups can be formed in proteins by the direct oxidation of the side chains from amino acids (threonine, lysine, proline, and arginine), oxidative cleavage of the peptide backbone via the α‐amidation pathway or via oxidation of glutamyl side chains; covalent binding to nonprotein compounds such as MDA from lipid oxidation could also generate carbonyl (Estévez, 2011). The effects of salt mixture on the carbonyl content of the restructured bacon are illustrated in Figure 1b. The carbonyl contents in the treatment groups (U1, U2, and U3) also increased significantly compared with the control group (U0) (*p* < .05), but the difference between 0.5% (0.25%KCl + 0.25%CaCl_2_) and 1.0% (0.5%KCl + 0.5%CaCl_2_) of salt mixture was insignificant (*p* > .05). The low‐level replacement of NaCl by KCl and CaCl_2_ could promote the oxidation of meat myofibrillar protein (Zheng, Han, Ge, Zhao, & Sun, 2019). The increased of ionic strength could change the assembly degree of myofibrillar protein (the major protein in meat) and induce a destabilized structure of the protein, resulting in an enhanced exposure to pro‐oxidants and, thus, promoting carbonyl formation (Liu, Xiong, & Chen, 2011). The free radicals produced by the UT can be contributed to the increased protein carbonyl contents in beef (Kang et al., 2016). In addition, some researchers have revealed that radicals and hydroperoxides formed as a result of lipid oxidation may attack vulnerable amino acid chains in protein molecules to generate carbonyl compounds (Estévez, 2011; Li et al., 2019; Wen et al., 2019). It has been implied that the accelerated protein oxidation in the restructured bacon formulated with salt mixtures could also be related to the enhanced lipid oxidation in these groups (Figure 1a). The present study showed that the salt mixture could promote the protein oxidation of the restructured bacon, but the oxidation degree of the bacon could reach saturation with the addition of 1.0% salt mixture. It implied that the protein oxidation contribution to the quality of the bacon was greater.

### 3.2 Free fatty acids

Free fatty acid formation is due to lipolysis; the composition of FFAs is one of the most important components that can influence the nutritional value and sensory properties, especially flavor, of meat products (Wood et al., 2008). As shown in Table 2, the percentage of FFAs in the control group (U0) presented the order as follows: monounsaturated fatty acids (MUFA)> saturated fatty acids (SFA)> polyunsaturated fatty acids (PUFA). Moreover, this order was not affected by the addition of salt mixture. These results denoted that the liberation of FFAs in all restructured bacon groups mainly originated from the triglycerides that are abundant in MUFA, which are present at high levels in the adipose tissue of the restructured bacon. It could be seen that the percentages of SFA and MUFA increased significantly (*p* < .05), while the percentage of PUFA decreased significantly (*p* < .05) in the all treatment groups (U1, U2, and U3) compared with the control (U0). With respect to the PUFA/SFA ratios, the nutritional recommendations are that this ratio should be approximately 0.45 (Wood et al., 2008). In this study, the ratios of PUFA/SFA approached the values recommended for the human diet (Table 2). The PUFA/SFA ratios were significantly decreased in the U1, U2, and U3 groups compared with the U0 group (*p* < .05). The composition of FFA is directly affected by lipid oxidation, and PUFA are more subject to oxidation than MUFA and SFA (Coutron‐Gambotti & Gandemer, 1999). According to present results of the TBARS values, it was speculated that salt mixture accelerated the oxidation of PUFA in the UT condition, thus inducing the decreased PUFA/SFA ratio. Moreover, the FFAs predominately found in the restructured bacon were C16:0 (palmitic), C18:0 (stearic), C18:1 (oleic), and C18:2 (n‐6) (linoleic), as found in dry‐cured loin (Armenteros, Aristoy, Barat, & Toldra, 2009) and low‐sodium dry fermented sausage (dos Santos et al., 2017). Those FFAs, which are the main sources of lipogenic volatile compounds, such as hexanal and nonanal, are commonly found at high concentrations in bacon (Huanget al., 2014).

**TABLE 2:** Effect of salt mixture on free fatty acid composition of the reduced‐sodium restructured bacon under ultrasound treatments (U0, U1, U2, and U3)*

FFA (% of total FFAs)	U0	U1	U2	U3
C10:0	0.051 ± 0.000^c^	0.053 ± 0.001^b^	0.054 ± 0.000^a^	0.053 ± 0.000^b^
C12:0	0.056 ± 0.000^b^	0.058 ± 0.001^a^	0.058 ± 0.001^a^	0.058 ± 0.000^a^
C14:0	1.167 ± 0.006^c^	1.200 ± 0.008^b^	1.219 ± 0.005^a^	1.198 ± 0.003^b^
C16:0	22.161 ± 0.089^d^	22.649 ± 0.039^b^	22.915 ± 0.046^a^	22.485 ± 0.028^c^
C16:1	1.868 ± 0.005^c^	1.908 ± 0.008^b^	1.938 ± 0.012^a^	1.927 ± 0.006^a^
C18:0	11.306 ± 0.085^b^	11.777 ± 0.170^a^	11.777 ± 0.216^a^	11.372 ± 0.173^b^
C18:1	45.637 ± 0.009^b^	46.146 ± 0.240^a^	46.359 ± 0.246^a^	46.307 ± 0.194^a^
C18:2 n6	14.802 ± 0.085^a^	13.487 ± 0.015^c^	13.082 ± 0.070^d^	13.944 ± 0.50^b^
C18:3 n3	0.799 ± 0.006^a^	0.710 ± 0.000^c^	0.676 ± 0.005^d^	0.747 ± 0.003^b^
C20:1 n9	1.050 ± 0.004^a^	1.076 ± 0.005^a^	1.258 ± 0.331^a^	1.068 ± 0.000^a^
C20:2 n6	0.875 ± 0.007^a^	0.809 ± 0.008^c^	0.786 ± 0.008^d^	0.841 ± 0.005^b^
ƩSFA	34.742 ± 0.154^c^	35.738 ± 0.121^a^	36.024 ± 0.261^a^	35.166 ± 0.145^b^
ƩMUFA	48.555 ± 0.013^b^	49.130 ± 0.253^a^	49.556 ± 0.315^a^	49.302 ± 0.193^a^
ƩPUFA	16.477 ± 0.095^a^	15.006 ± 0.023^c^	14.544 ± 0.083^d^	15.532 ± 0.056^b^
PUFA/SFA	0.474 ± 0.002^a^	0.420 ± 0.001^c^	0.404 ± 0.004^d^	0.442 ± 0.001^b^

Note

Different superscript letters (^a–d^) in the same row indicate statistically significant differences at *p* < .05 (*n* = 3).

* U0: 1.5%NaCl + UT 600 w; U1: 1.5%NaCl + 0.125%KCl + 0.125%CaCl_2_ + UT 600 w; U2: 1.5%NaCl + 0.25%KCl + 0.25%CaCl_2_ + UT 600 w; U3: 1.5%NaCl + 0.50%KCl + 0.50%CaCl_2_ + UT 600 w.

### 3.3 Free amino acids

Free fatty acids, the final products of proteolysis, have been considered to be of significance not only for their contribution to specific taste but also for their participation in the generation of volatile compounds that provide the flavor in meat products (Toldrá & Flores, 1998). As presented in Table 3, the major FAA in each restructured bacon group was His, followed by Ala and Thr. The salt mixture in the addition levels of U1 and U2 could significantly increase the content of total FAA (*p* < .05). KCl and CaCl_2_ play important roles in regulating (activation/inhibition) the activity of aminopeptidase enzymes in muscle and exert an effect on the release of FAA (Armenteros et al., 2009). In the present study, KCl and CaCl_2_ might activate aminopeptidase enzymes, thereby contributing to the generation of FAA. The total FAA in the U3 group was significantly decreased in relation to the U2 group, which might be attributed to inhibition of aminopeptidase enzyme activity at higher salt concentrations. With respect to single FAA, it was observed that hydrophobic amino acids (such as Ala, Val, and Ile) were significantly increased with the addition of chloride salt mixtures. Protein oxidation induced by KCl and CaCl_2_ could cause an unfolding process of protein and further promoted the exposure of hydrophobic groups, which likely facilitate the recognition of proteins by proteases to generate more FAA (Wen et al., 2019). During the curing stage, the solubility of salt‐soluble protein is positively related to the environmental ionic strength (Andreetta‐Gorelkina, Greiff, Rustad, & Aursand, 2016), and the interactions of salt ions (Na+, K+, Ca2+, and Cl‐) can facilitate the specificity of proteolytic enzymes for peptide bonds (Gan et al., 2019), thus promoting the degradation of peptide bonds. The increase of FAA results from the degradation of peptide bonds and proteins, and its decrease can be attributed to Strecker and Maillard reactions (Qi, Liu, Zhou, & Xu, 2017). These observations implied that the significant increases of FAA content in U1 and U2 group (*p* < .05) could be attributed to the promoted degradation of peptide bonds and proteins when the salt mixture level was 0.25% (0.125%KCl + 0.125%CaCl_2_) or 0.5% (0.25%KCl + 0.25%CaCl_2_), and the significant decrease of FAA content in the U3 group (*p* < .05) could have resulted from the high solubility of proteins and both fast reactions of FAA when the salt mixture level was 1.0% (0.5%KCl + 0.5%CaCl_2_).

**TABLE 3:** Effect of salt mixture on free amino acid composition of the reduced‐sodium restructured bacon under ultrasound treatments (U0, U1, U2, and U3)*

FAA (mg/100g)	U0	U1	U2	U3
Gly	8.55 ± 0.02^b^	9.31 ± 0.08^a^	9.50 ± 0.16^a^	8.66 ± 0.24^b^
IIe	2.99 ± 0.04^c^	3.38 ± 0.05^b^	3.53 ± 0.09^a^	3.07 ± 0.01^c^
Leu	5.74 ± 0.24^c^	7.06 ± 0.24^ab^	7.23 ± 0.45^a^	6.55 ± 0.05^b^
Ala	18.96 ± 0.07^b^	19.85 ± 0.28^a^	20.07 ± 0.36^a^	18.97 ± 0.34^b^
Val	4.54 ± 0.02^c^	6.37 ± 0.07^a^	6.53 ± 0.15^a^	5.47 ± 0.78^b^
Phe	4.25 ± 0.85^a^	6.05 ± 1.34^a^	6.11 ± 1.42^a^	6.12 ± 0.02^a^
Pro	4.81 ± 0.27^bc^	5.12 ± 0.40^ab^	5.45 ± 0.16^a^	4.54 ± 0.13^c^
Met	2.08 ± 0.03^c^	3.33 ± 0.05^a^	3.46 ± 0.10^a^	2.83 ± 0.46^b^
Ser	5.85 ± 0.08^b^	6.77 ± 0.09^a^	6.77 ± 0.17^a^	5.84 ± 0.04^b^
Thr	9.99 ± 0.29^a^	10.20 ± 0.11^a^	10.15 ± 0.45^a^	9.15 ± 0.16^b^
Tyr	2.59 ± 0.91^a^	2.35 ± 0.44^a^	2.41 ± 0.50^a^	2.57 ± 0.05^a^
Arg	5.35 ± 0.07^c^	6.59 ± 0.12^a^	6.61 ± 0.17^a^	5.88 ± 0.05^b^
Asp	0.37 ± 0.21^b^	0.71 ± 0.01^a^	0.92 ± 0.13^a^	0.44 ± 0.01^b^
His	58.73 ± 1.23^c^	60.77 ± 0.97^ab^	62.26 ± 1.31^a^	59.21 ± 0.26^bc^
Glu	8.26 ± 0.02^c^	9.15 ± 0.10^b^	9.62 ± 0.18^a^	7.91 ± 0.02^d^
Lys	5.86 ± 0.26^c^	6.59 ± 0.15^ab^	6.67 ± 0.33^a^	6.16 ± 0.23^bc^
Total FAA	148.96 ± 1.89^b^	163.60 ± 0.22^a^	167.30 ± 4.75^a^	153.38 ± 1.55^b^

Note

Different superscript letters (^a–d^) in the same row indicate statistically significant differences at *p* < .05 (*n* = 3).

* U0: 1.5%NaCl + UT 600 w; U1: 1.5%NaCl + 0.125%KCl + 0.125%CaCl_2_ + UT 600 w; U2: 1.5%NaCl + 0.25%KCl + 0.25%CaCl_2_ + UT 600 w; U3: 1.5%NaCl + 0.50%KCl + 0.50%CaCl_2_ + UT 600 w.

### 3.4 Volatile flavor compounds

#### 3.4.1 Volatile flavor compounds

As shown in Table 4, the 61 kinds of volatile flavor compounds identified in the restructured bacon were grouped by six different chemical families, including 11 phenols, 7 aldehydes, 17 ketones, 14 hydrocarbons, 3 alcohols, and 9 heterocycles. In total, 45, 46, 46, and 49 volatile flavor compounds were identified in the U0, U1, U2, and U3 groups, respectively. The generation of most volatile flavor compounds in cooked meat products is related to several reactions that take place in the meats, such as lipid oxidation, Maillard reaction, vitamin degradation, and the interaction between Maillard reaction products and lipid‐oxidized products (van Ba, Hwang, Jeong, & Touseef, 2012). The amounts of total volatile flavor compounds (between 843.68 and 994.91 AU × 10^5^) in the treatment groups (U1, U2, and U3) were higher than that (668.20 AU × 10^5^) in the control group (U0). It was reasonable to speculate that the addition of KCl and CaCl_2_ could promote the formation of volatile flavor compounds in the restructured bacon. The effect of each type of volatile flavor compound with the addition of salt mixture is further discussed below.

**TABLE 4:** Effect of salt mixture on volatile composition (AU*10^5^) of the reduced‐sodium restructured bacon under ultrasound treatments (U0, U1, U2, and U3)*

Compounds	Threshold value (μg/kg)	U0	U1	U2	U3
Phenols					
Phenol	5,501	46.95 ± 1.86^c^	52.00 ± 5.39^bc^	58.79 ± 0.46^a^	55.43 ± 1.94^ab^
o‐Cresol	45	21.24 ± 1.15^b^	23.06 ± 1.56^b^	27.48 ± 0.22^a^	25.80 ± 0.94^a^
m‐Cresol	31	22.75 ± 1.38^b^	7.36 ± 1.24^c^	26.68 ± 1.25^a^	25.25 ± 0.91^a^
2‐Methoxy‐phenol	3	123.95 ± 4.98^b^	134.70 ± 10.09^b^	156.60 ± 2.84^a^	147.65 ± 5.51^a^
2,6‐Xylenol	400	3.72 ± 0.89^ab^	2.69 ± 0.12^b^	3.84 ± 0.14^a^	3.20 ± 0.53^ab^
3,5‐Xylenol	5,000	5.52 ± 0.39^ab^	6.31 ± 0.71^a^	5.94 ± 0.24^ab^	5.23 ± 0.23^b^
3‐Ethylphenol	140	2.45 ± 0.04^c^	2.48 ± 0.15^c^	2.83 ± 0.07^b^	3.18 ± 0.32^a^
2‐Methoxy‐5‐methylphenol	13	4.46 ± 0.12^c^	4.64 ± 0.34^bc^	5.41 ± 0.10^a^	4.97 ± 0.29^ab^
2‐Methoxy‐4‐methylphenol	21	57.52 ± 2.83^b^	61.62 ± 3.87^ab^	66.69 ± 2.95^a^	61.78 ± 2.76^ab^
3,4‐Dimethoxyphenol	–	n.d.	n.d.	0.77 ± 0.12	n.d.
2,6‐Dimethoxyphenol	263	5.04 ± 0.68^b^	6.69 ± 0.52^a^	4.55 ± 0.47^bc^	4.07 ± 0.10^c^
Subtotal		293.60 ± 13.50^b^	301.55 ± 22.46^b^	359.58 ± 7.10^a^	336.55 ± 13.53^a^
Aldehydes					
Hexanal	5	6.02 ± 0.20^c^	10.48 ± 2.57^b^	13.79 ± 1.56^a^	11.68 ± 0.65^ab^
Furfural	3,000	98.58 ± 6.13^d^	201.91 ± 14.82^c^	254.37 ± 18.98^b^	300.25 ± 19.48^a^
5‐Methyl furfural	1,110	38.12 ± 1.45^c^	49.36 ± 2.95^b^	57.37 ± 1.59^a^	57.44 ± 2.31^a^
Nonanal	1.1	16.94 ± 0.69^b^	23.82 ± 6.61^a^	30.59 ± 2.40^a^	30.53 ± 1.06^a^
Decanal	0.2	3.93 ± 0.09^a^	0.77 ± 0.12^c^	1.26 ± 0.20^b^	0.75 ± 0.04^c^
Neral	53	0.20 ± 0.10	n.d.	n.d.	n.d.
Citral	120	n.d.	0.46 ± 0.12	n.d.	n.d.
Subtotal		163.80 ± 6.42^d^	286.81 ± 22.87^c^	357.38 ± 23.74^b^	400.64 ± 22.24^a^
Ketones					
Cyclopentanone	9,300	2.46 ± 0.20^b^	2.59 ± 0.40^b^	3.63 ± 0.09^ab^	4.76 ± 1.50^a^
1‐Acetoxyacetone	–	n.d.	n.d.	n.d.	0.58 ± 0.03
2‐Octanone	50	5.37 ± 0.52^a^	n.d.	4.17 ± 0.68^b^	4.01 ± 0.74^b^
2‐Hexanone	560	n.d.	3.87 ± 0.99	n.d.	n.d.
2‐Methylcyclopentenone	–	11.91 ± 1.02^b^	13.37 ± 1.16^b^	18.59 ± 2.01^a^	17.75 ± 2.27^a^
Acetonylacetone	–	2.06 ± 0.74^a^	2.02 ± 0.87^a^	1.78 ± 0.26^a^	1.93 ± 0.08^a^
3,4‐Dimethylcyclopentenone	–	3.00 ± 0.49^a^	3.48 ± 1.34^a^	3.74 ± 0.56^a^	3.11 ± 0.48^a^
3‐Methyl‐3‐cyclohexen‐1‐one	–	2.43 ± 0.20^b^	2.75 ± 0.10^a^	n.d.	2.89 ± 0.01^a^
2‐Methyl‐2‐propynyl‐2‐acetone	–	1.23 ± 0.34^a^	n.d.	1.27 ± 0.16^a^	n.d.
2,3‐Octanedione	–	n.d.	n.d.	n.d.	1.26 ± 0.05
Methyl cyclopentenolone	300	n.d.	9.22 ± 0.79	n.d.	n.d.
2,3‐Dimethyl‐2‐cyclopentenone	–	14.59 ± 0.46^d^	18.05 ± 1.15^c^	25.22 ± 1.24^a^	21.30 ± 1.21^b^
3‐Ethyl‐2‐cyclopenten−1‐one	–	5.72 ± 0.34^b^	0.44 ± 0.02^c^	7.65 ± 0.56^a^	7.16 ± 0.38^a^
Mesityl oxide	–	0.38 ± 0.00^b^	n.d.	0.62 ± 0.05^a^	0.54 ± 0.12^a^
4,4‐Dimethyl‐2‐cyclohexen‐1‐one	–	0.60 ± 0.07^a^	0.55 ± 0.10^a^	0.62 ± 0.06^a^	0.52 ± 0.02^a^
cis‐Hexahydro‐7a‐methyl‐2(3H)‐benzofuranone	–	n.d.	n.d.	0.22 ± 0.05	n.d.
Siro[5.5]undecane‐5,11‐dione	–	n.d.	n.d.	n.d.	0.48 ± 0.26
Subtotal		49.76 ± 3.51^b^	56.35 ± 5.26^b^	67.52 ± 4.24^a^	66.31 ± 6.96^a^
Hydrocarbons					
Propan‐2‐ylidenecyclopentane	–	n.d.	n.d.	3.19 ± 0.25	n.d.
1‐Acetylcyclohexene	–	n.d.	1.70 ± 0.13^a^	n.d.	0.59 ± 0.12^b^
2,2,5‐Trimethyldecane	–	n.d.	13.07 ± 0.90^a^	16.99 ± 2.51^a^	18.15 ± 4.70^a^
3,4‐Dimethylhexa‐2,4‐diene	–	n.d.	n.d.	4.89 ± 0.20^b^	5.34 ± 0.31^a^
Propan‐2‐ylidenecyclohexane	–	2.85 ± 0.12^c^	3.19 ± 0.20^bc^	4.06 ± 0.04^a^	3.55 ± 0.31^b^
3‐Methylundecane	–	1.64 ± 0.11^c^	1.83 ± 0.17^c^	2.39 ± 0.13^b^	2.88 ± 0.16^a^
Bis‐(R,R)1,1‐(1,2‐dimethyl‐1,2‐ethylene)cyclohexane	–	0.55 ± 0.03^a^	0.51 ± 0.10^a^	0.54 ± 0.04^a^	0.55 ± 0.01^a^
3,4‐Dimethoxytoluene	–	1.41 ± 0.03^b^	1.41 ± 0.13^b^	1.74 ± 0.03^a^	1.77 ± 0.18^a^
Tridecane	–	n.d.	n.d.	n.d.	0.54 ± 0.04
Tetradecane	1,000	0.37 ± 0.02^b^	0.39 ± 0.14^b^	0.61 ± 0.09^a^	n.d.
3‐Methyltridecane	–	0.71 ± 0.05^ab^	0.74 ± 0.11^ab^	0.65 ± 0.09^b^	0.85 ± 0.04^a^
Pentadecane	–	n.d.	1.01 ± 0.08	n.d.	n.d.
Hexadecane	–	1.05 ± 0.07^c^	n.d.	1.80 ± 0.10^a^	1.48 ± 0.01^b^
1,2,3‐Trimethoxybenzene	–	1.53 ± 0.15^a^	1.10 ± 0.06^bc^	1.27 ± 0.21^b^	0.98 ± 0.05^c^
Subtotal		10.11 ± 0.20^c^	24.95 ± 1.19^b^	38.14 ± 2.76^a^	36.68 ± 5.59^a^
Alcohols					
2‐Furanmethanol	4,500	8.67 ± 0.29	n.d.	n.d.	n.d.
3‐Furancarbinol	2,000	n.d.	8.18 ± 0.71^a^	n.d.	4.35 ± 0.31^b^
Linalool	37	0.87 ± 0.08^a^	0.35 ± 0.03^b^	0.32 ± 0.25^b^	0.15 ± 0.05^b^
Subtotal		9.55 ± 0.22^a^	8.53 ± 0.72^b^	0.32 ± 0.25^d^	4.50 ± 0.36^c^
Heterocycles and others					
3‐Methylpyridine	–	3.10 ± 0.21^a^	2.60 ± 0.31^a^	3.37 ± 0.32^a^	4.14 ± 1.88^a^
2‐Acetylfuran	15,025	39.44 ± 1.85^d^	121.20 ± 9.33^b^	145.50 ± 4.64^a^	55.41 ± 4.80^c^
3,5‐Dimethylpyridine	–	1.62 ± 0.33^a^	2.26 ± 1.43^a^	1.80 ± 0.13^a^	n.d.
3,4‐Lutidine	–	n.d.	n.d.	n.d.	1.15 ± 0.06
2‐Propionylfuran	–	5.13 ± 0.10^c^	5.21 ± 0.30^c^	6.81 ± 0.12^a^	6.15 ± 0.29^b^
2‐Acetyl‐5‐methylfuran	–	4.23 ± 0.07^b^	4.58 ± 0.50^b^	5.85 ± 0.52^a^	6.10 ± 0.45^a^
3‐Acetoxypyridine	–	82.20 ± 6.33^a^	23.49 ± 1.49^c^	n.d.	71.82 ± 1.13^b^
2‐Methyl‐5‐propionylfuran	–	0.48 ± 0.14^a^	0.45 ± 0.07^a^	0.44 ± 0.04^a^	0.36 ± 0.11^a^
1,2‐Dimethoxybenzene	–	5.17 ± 1.13^a^	5.69 ± 0.48^a^	6.10 ± 0.30^a^	5.10 ± 0.04^a^
Subtotal		141.39 ± 5.31^b^	165.48 ± 10.61^a^	169.87 ± 4.63^a^	150.22 ± 8.57^b^
Total		668.20 ± 19.70^c^	843.68 ± 61.12^b^	992.81 ± 22.63^a^	994.91 ± 57.24^a^

Note

Different superscript letters (^a–d^) in the same row indicate statistically significant differences at *p* < .05 (*n* = 3). AU: area units resulting of counting the total ion chromatogram (TIC) for each compound. –: not queried; n.d.: not detected.

* U0: 1.5%NaCl + UT 600 w; U1: 1.5%NaCl + 0.125%KCl + 0.125%CaCl_2_ + UT 600 w; U2: 1.5%NaCl + 0.25%KCl + 0.25%CaCl_2_ + UT 600 w; U3: 1.5%NaCl + 0.50%KCl + 0.50%CaCl_2_ + UT 600 w.

Phenols were the most abundant group of compounds in all restructured bacon groups (Table S1). Phenolic compounds (phenols and metoxyphenols) are the main contributor to the unique aroma and taste of smoked meat products (Petričević, Radovčić, Lukić, Listeš, & Medić, 2018). In the present study, the primary phenols in the restructured bacon were 2‐methoxy‐phenol, phenol, 2‐methoxy‐4‐methylphenol, o‐cresol, and m‐cresol. The other phenols identified in the restructured bacon included 2‐methoxy‐5‐methylphenol, o‐cresol with a phenolic odor, 2,6‐xylenol, 3,5‐xylenol, 3‐ethylphenol, 3,4‐dimethoxyphenol, and 2,6‐dimethylphenol. Most of the phenols in our findings have also been identified in other smoked meat products, such as Hungarian‐type salami, Croatian dry‐cured hams, and smoked cooked loin (Kosowska et al., 2018; Petričević et al., 2018; Söllner & Schieberle, 2009). The amounts of total phenols in U2 and U3 groups were significantly increased compared with the U0 group (*p* < .05).

Aldehydes were the second most abundant group of compounds in the restructured bacon (Table S1), among which linear aldehydes result from the oxidation of unsaturated fatty acids and branched aldehydes from amino acid degradation. Moreover, most aldehydes play a major role in developing the flavor of meat products due to their low odor threshold values (Estévez, 2011; Petričević et al., 2018; Wen et al., 2019). The contents of hexanal and nonanal were significantly increased when salt mixture was added, and they reached the maximum values in the U2 group. It has been reported that the content of volatile flavor compounds derived from lipid oxidation in fermented sausages was positively correlated with the TBARS values (Olivares, Navarro, & Flores, 2011). Therefore, the increased levels of linear aldehydes in the restructured bacon formulated with salt mixture were likely related to the increased TBARS values (Figure 1a). Furfural and 5‐methyl furfural are generated by the Maillard reaction (Wen et al., 2019), and the contents of the two aldehydes were significantly increased in the treatments with salt mixtures compared to the control (*p* < .05); the effect was generally consistent with carbonyl content results (Figure 1b). Due to the high odor threshold value, the two aldehydes could contribute less to the restructured bacon than the linear aldehydes.

Ketones are mainly derived from the thermal oxidation of PUFA and the degradation of amino acids, contributing to the flavor of meat products with fruity and fragrant aromas (Zhang et al., 2019). The total contents of the ketones in the U2 and U3 groups were significantly increased compared with the control (U0) (*p* < .05). According to Saldaña et al. (2019), methyl ketones come from lipid oxidation; KCl and CaCl_2_ may promote lipid oxidation, resulting in the increased formation of methyl ketones such as 2‐methyl‐2‐propynyl‐2‐acetone and mesityl oxide. Cyclopentones are typical volatiles of wood smoke (del Olmo, Calzada, & Nuñez, 2014). Some cyclopentones, such as cyclopentanone and 2,3‐dimethyl‐2‐cyclopentenone, were increased with the treatment of salt mixture (*p* < .05), which could be attributed to the actions of KCl and CaCl_2_ in enhancing the diffusion of liquid smoke, leading to an increased release of these ketones.

Hydrocarbons originate from the thermal degradation of lipids through thermal homolysis or autoxidation of long‐chain fatty acids (Domínguez, Gómez, Fonseca, & Lorenzo, 2014). The hydrocarbon contents in three treatment groups (U1, U2, and U3) were increased significantly compared with the control (U0) (*p* < .05). These results could be ascribed to the lipid oxidation promoted by the salt mixture. However, hydrocarbons are generally considered as having no substantial influence on the flavor of meat products due to their relatively high odor threshold values (del Olmo et al., 2014).

Alcohols mainly arise from the reaction products of lipid oxidation (Petričević et al., 2018). Similar to hydrocarbons, alcohols are generally not thought of as important to contributing flavor to meat products because of their high odor thresholds (Domínguez et al., 2014). 2‐Furanmethanol was found in U0, while it was not present in the U1, U2, and U3. Additionally, levels of linalool were significantly decreased with the addition of salt mixture (*p* < .05).

Heterocycle compounds are formed by the Maillard reaction and provide important contributions to sweet, caramel, and barbecue‐like notes, leading to the improved flavor of meat products (Romero, Raghavan, & Ho, 2013; Zhang et al., 2019). Of the heterocycle compounds, furans are usually generated through the heating process. The levels of most of the furans (such as 2‐acetylfuran and 2‐propionylfuran) were higher in the treated groups (U2 and U3) compared with the control group (U0). The heterocycle and carbonyl contents showed similar variation tendencies, and it could be considered that the change in heterocycle content was due to the promotion of protein oxidation by the salt mixture.

#### 3.4.2 Relative odor activity value

Due to the variety of volatile flavor compounds and their different threshold values, the content of each compound cannot reflect the true contribution to the whole aroma profile. Therefore, ROAV was used to evaluate the contribution of volatile flavor compounds in the whole aroma profile. The compounds with ROAV ≥ 1 were considered as the key volatile flavor compounds in the restructured bacon. The higher ROAV was, the higher the degree of compound contribution was to the flavor. The compounds with 0.1 ≤ ROAV < 1 were considered to be potential flavor compounds (Zhuang et al., 2016). As shown in Table 5, the types of key volatile compounds in all restructured bacon groups were the same, including four phenols (o‐cresol, m‐cresol, 2‐methoxy‐phenol, 2‐methoxy‐4‐methylphenol) and three aldehydes (hexanal, nonanal, and decanal). The addition of salt mixture could significantly increase the release of the four phenols, especially 2‐methoxy‐phenol. The relative contents of 2‐methoxy‐phenol were the highest of the total volatile compounds (Table 4). In agreement with the present study, 2‐methoxy‐phenol had the highest content in bacon and was essential for the sensory characteristics of smoked meat products (Saldaña et al., 2019). The total contents of the three aldehydes in the treatment groups (U1, U2, and U3) increased significantly compared with the control (U0) (*p* < .05) and reached the highest values at the 0.5% addition level of salt mixture. Due to the low odor threshold value, those aldehydes could promote flavor in the restructured bacon. The hexanal content and TBARS values showed similar variation tendencies, but the decanal content and TBARS values were exactly the opposite. Three kinds of potential volatile flavor compounds (2‐methoxy‐5‐methylphenol, furfural, and 5‐methyl furfural) provide fewer contributions to the restructured bacon compared with the key volatile flavor compounds due to their high odor threshold values, although their contents were high. It could be concluded that salt mixture did not affect the types of key volatile flavor compounds and could promote the release of key phenols and the formation of key aldehydes in the restructured bacon.

**TABLE 5:** The ROAVs of major volatile flavor compounds of the reduced‐sodium restructured bacon under ultrasound treatment with different salt mixture content (U0, U1, U2, and U3)*

Compounds	Threshold value (μg/kg)	U0	U1	U2	U3
o‐Cresol	45	1.14	1.14	1.17	1.16
m‐Cresol	31	1.78	0.53	1.65	1.65
2‐Methoxy‐phenol	3	100.00	100.00	100.00	100.00
2‐Methoxy‐4‐methylphenol	21	6.63	6.54	6.08	5.98
Hexanal	5	2.92	4.67	5.28	4.75
Nonanal	1.1	37.27	48.23	53.27	56.39
Decanal	0.2	47.54	8.57	12.05	7.64
2‐Methoxy‐5‐methylphenol	13	0.83	0.79	0.80	0.78
Furfural	3,000	0.08	0.15	0.16	0.20
5‐Methyl furfural	1,110	0.08	0.10	0.10	0.11

Note

Other volatile compounds (ROAV < 0.1) are not included in the table.

* U0: 1.5%NaCl + UT 600 w; U1: 1.5%NaCl + 0.125%KCl + 0.125%CaCl_2_ + UT 600 w; U2: 1.5%NaCl + 0.25%KCl + 0.25%CaCl_2_ + UT 600 w; U3: 1.5%NaCl + 0.50%KCl + 0.50%CaCl_2_ + UT 600 w.

### 3.5 Sensory analysis

As shown in Table 6, for the attribute aroma, the U2 group showed a significantly higher score than the other groups (U0 and U1) (*p* < .05). This was probably because the 0.5% salt mixture promoted lipid and protein oxidation and increased the contents of the key flavor compounds in the restructured bacon, generating better aroma in the final product. This finding was similar to the results of low‐salt salami containing 0.25% KCl and 0.25% CaCl_2_ (de Almeida et al., 2016). The taste of the restructured bacon exhibited a similar changing trend to the aroma when salt mixture was added. The increase in the taste score in the U2 group could be attributed to the increased generation of umami amino acids (Asp, Glu, and Gly) (Dashdorj, Amna, & Hwang, 2015) with the addition of the 0.5% salt mixture (Table 3). The texture of the U3 restructured bacon had a significantly lower score than the other groups (U0, U1, and U2) (*p* < .05). The additions of KCl and CaCl_2_ cause texture deterioration in dry‐cured ham (Armenteros et al., 2012) and salami (de Almeida et al., 2016), respectively. It is possible that the addition of excessive CaCl_2_ can change the structure of meat, providing an open access in the meat matrix and increasing the release of water in meat products (Vidal et al., 2019), which may impact the texture. There were no significant differences between any of the treatments regarding color and aftertaste. In the present study, the overall quality was significantly (*p* < .05) improved in group U2 compared with the other groups (U0, U1, and U3).

**TABLE 6:** The sensory scores of the reduced‐sodium restructured bacon under ultrasound treatment with different salt mixture content (U0, U1, U2, and U3)*

	U0	U1	U2	U3
Aroma	6.40 ± 0.82^b^	6.40 ± 1.14^b^	7.90 ± 0.89^a^	6.90 ± 1.14^ab^
Taste	6.60 ± 1.14^ab^	6.30 ± 1.10^b^	7.80 ± 0.76^a^	7.50 ± 1.00^ab^
Color	6.90 ± 0.74^a^	7.00 ± 0.71^a^	6.90 ± 1.24^a^	6.80 ± 0.57^a^
Texture	7.10 ± 1.14^a^	7.10 ± 0.89^a^	7.00 ± 0.79^a^	5.80 ± 0.57^b^
Aftertaste	7.50 ± 0.50^a^	7.00 ± 0.79^a^	7.40 ± 0.82^a^	6.90 ± 0.89^a^
Overall quality	6.80 ± 0.41^b^	6.62 ± 0.54^b^	7.58 ± 0.49^a^	6.96 ± 0.27^b^

Note

Different superscript letters (^a–b^) in the same row indicate statistically significant differences at *p* < .05 (*n* = 9).

* U0: 1.5%NaCl + UT 600 w; U1: 1.5%NaCl + 0.125%KCl + 0.125%CaCl_2_ + UT 600 w; U2: 1.5%NaCl + 0.25%KCl + 0.25%CaCl_2_ + UT 600 w; U3: 1.5%NaCl + 0.50%KCl + 0.50%CaCl_2_ + UT 600 w.

### 3.6 Principal component analysis (PCA)

**FIGURE 2:**
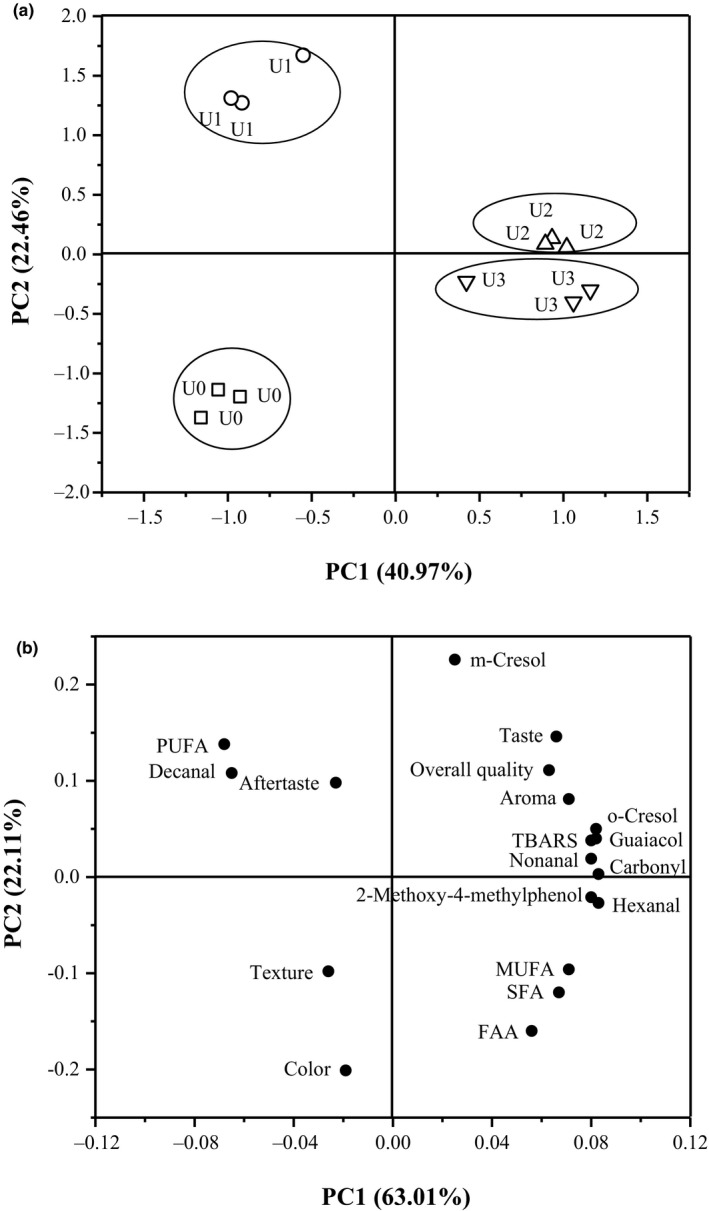
The PCA loading plot (a) for volatile compounds of the reduced‐sodium restructured bacon under ultrasound treatment with difference treatment (*n* = 3); The PCA loading plot (b) of key volatile compounds, lipid oxidation, protein oxidation, FFAs, FAA, and sensory evaluation of the reduced‐sodium restructured bacon under ultrasound treatment. *U0: 1.5%NaCl + UT 600 w; U1: 1.5%NaCl + 0.125%KCl + 0.125%CaCl_2_ + UT 600 w; U2: 1.5%NaCl + 0.25%KCl + 0.25%CaCl_2_ + UT 600 w; U3: 1.5%NaCl + 0.50%KCl + 0.50%CaCl_2_ + UT 600 w

Principal component analysis was used to explore the impacts of volatile flavor compounds of the restructured bacon with different treatments and the relationships among the key volatile flavor compounds, lipid oxidation, protein oxidation, FFA, FAA, and sensory evaluation. As shown in Figure 2a, the first principal component (PC1) and second principal component (PC2) explained 63.43% of the total variance (40.97% for PC1 and 22.46% for PC2). The results of the PCA showed a good discrimination of the groups with different salt mixture contents. The major variation resulting from PC1 was caused by the difference between the low‐ and high‐level salt mixture contents. This result indicated that the addition of salt mixture had a major influence on the volatile flavor compounds of the restructured bacon, but the effect was weaker between groups U2 and U3. This finding could be related to the fact that there were no significant differences between the contents of total volatile flavor compounds in the U2 and U3 groups (*p* > .05). As shown in Figure 2b, PC1 and PC2 explained 85.12% of the total variance (63.01% for PC1, and 22.11% for PC2). It was apparent that aroma, taste, and overall quality were positively correlated with the TBARS, carbonyl, MUFA, SFA, FAA, and six kinds of key volatile compounds (m‐cresol, o‐cresol, guaiacol, nonanal, hexanal, and 2‐methoxy‐4‐methylphenol), indicating that the flavor of the restructured bacon was related to the oxidation of lipids and proteins and that the enhancement of its flavor was attributed to increases in key volatile flavor compounds.

### 3.7 Comprehensive discussion

As indicated above, the salt mixture does not affect the types of key volatile flavor compounds in the restructured bacon (Table 5). The mixture of 0.25% KCl and 0.25% CaCl_2_ could significantly promote the release of key phenol volatile flavor compounds and the formation of key aldehyde volatile flavor compounds (*p* < .05) (Table 4, Table S2). Therefore, the aroma and overall quality were significantly enhanced in the U2 group (*p* < .05) (Table 6). The key phenol volatile flavor compounds may mainly come from liquid smoke, which produces a smoky flavor and can greatly contribute to bacon flavor due to its low odor threshold (Petričević et al., 2018). The three key aldehyde volatile flavor compounds belong to linear aldehydes and mainly result from unsaturated fatty acid oxidation (Wen et al., 2019). For example, hexanal is a product of linoleic acid oxidation, and decanal is produced in the oxidation of oleic acid; these aldehydes play an important role in the overall flavor of meat products due to their low odor thresholds (Zhuang et al., 2016). The salt mixture could promote the formation of linear aldehydes from the oxidation of PUFA, which was beneficial for improving the flavor of the restructured bacon (Table 3). The potential volatile flavor compounds (furfural and 5‐methyl furfural) are generated by the Maillard reaction (Wen et al., 2019). The salt mixture could significantly improve the formation of these compounds (*p* < .05), and the change was generally consistent with the results of carbonyl content (Figure 1b). It was suggested that the increases in two potential volatile aldehydes could be related to the promotion of protein oxidation in the restructured bacon by the salt mixture. Therefore, these results showed that the mixture of 0.25% KCl and 0.25% CaCl_2_ could significantly enhance the lipid and protein oxidation of the restructured bacon (*p* < .05) with UT and could improve flavor of the restructured bacon (*p < *.05) by promoting the release of major volatile phenolic compounds and the formation of major volatile aldehyde compounds.

## 4 CONCLUSION

The mixture of 0.25% KCl and 0.25% CaCl_2_ could significantly improve the flavor of reduced‐sodium restructured bacon with ultrasound treatment (600 w) (*p < *.05) by promoting the release of five major volatile phenolic compounds (o‐cresol, m‐cresol, 2‐methoxy‐phenol, 2‐methoxy‐4‐methylphenol, and 2‐methoxy‐5‐methylphenol) and the formation of five major volatile aldehyde compounds (hexanal, nonanal, decanal, furfural, and 5‐methyl furfural). The increases in major volatile aldehyde compounds of the UT‐restructured bacon were due to the promotion of lipid oxidation (*p* < .05) by the addition of the salt mixture. These findings implied that the combination of salt mixtures and UT could be used as a promising technique for the flavor improvement of reduced‐sodium products in the meat industry.

## CONFLICT OF INTEREST

The authors declare no competing financial interest.

## Supporting information

Table S1‐S3
