# Supplementary material for: Effect of salt mixture on flavor of reduced‐sodium restructured bacon with ultrasound treatment
Source: Food Sci Nutr. 2020 May 29;8(7):3857–71. doi: 10.1002/fsn3.1679 (PMC7382110; doi:10.1002/fsn3.1679)
Supplement: Supplementary file 1 — Table S1‐S3 [file FSN3-8-3857-s001.docx]

**Supplementary material**

**TABLE S1. Effect of salt mixture on various chemical families of volatile flavor compounds in reduced-sodium restructured bacon with ultrasound treatments (U0, U1, U2 and U3)** *

| Compounds / % | U0 | U1 | U2 | U3 |
| --- | --- | --- | --- | --- |
| Phenols | 43.93±0.88^a^ | 35.74±0.77^bc^ | 36.24±1.54^b^ | 33.85±0.61^c^ |
| Aldehydes | 24.52±0.79^d^ | 33.99±0.57^c^ | 35.97±1.57^b^ | 40.27±0.08^a^ |
| Ketones | 7.45±0.55^a^ | 6.68±0.27^b^ | 6.80±0.33^ab^ | 6.65±0.33^b^ |
| Hydrocarbons | 1.51±0.05^c^ | 2.96±0.08^b^ | 3.84±0.25^a^ | 3.67±0.36^a^ |
| Alcohols | 1.43±0.02^a^ | 1.01±0.07^b^ | 0.03±0.02^d^ | 0.45±0.01^c^ |
| Heterocycles and others | 21.16±0.70^a^ | 19.62±0.17^b^ | 17.00±0.47^c^ | 15.10±0.01^d^ |

^a–b^ Different letters in the same row indicate statistically significant differences at P <0.05 (n=3).

* U0: 1.5%NaCl + UT 600 w; U1: 1.5%NaCl + 0.125%KCl + 0.125%CaCl_2_ + UT 600 w; U2: 1.5%NaCl + 0.25%KCl + 0.25%CaCl_2_ + UT 600 w; U3: 1.5%NaCl + 0.50%KCl + 0.50%CaCl_2_ + UT 600 w.

**TABLE S2. Effect of salt mixture on major volatile flavor compounds of reduced-sodium restructured bacon with ultrasound treatments (U0, U1, U2 and U3)** *

| compounds/ AU*10^5^ | | U0 | U1 | U2 | U3 |
| --- | --- | --- | --- | --- | --- |
| A | o-Cresol | 21.24±1.15^b^ | 23.06±1.56^b^ | 27.48±0.22^a^ | 25.80±0.94^a^ |
|  | m-Cresol | 22.75±1.38^b^ | 7.36±1.24^c^ | 26.68±1.25^a^ | 25.25±0.91^a^ |
|  | 2-Methoxy-phenol | 123.95±4.98^b^ | 134.70±10.09^b^ | 156.60±2.84^a^ | 147.65±5.51^a^ |
|  | 2-Methoxy-4-methylphenol | 57.52±2.83^b^ | 61.62±3.87^ab^ | 66.69±2.95^a^ | 61.78±2.76^ab^ |
|  | Hexanal | 6.02±0.20^c^ | 10.48±2.57^b^ | 13.79±1.56^a^ | 11.68±0.65^ab^ |
|  | Nonanal | 16.94±0.69^b^ | 23.82±6.61^a^ | 30.59±2.40^a^ | 30.53±1.06^a^ |
|  | Decanal | 3.93±0.09^a^ | 0.77±0.12^c^ | 1.26±0.20^b^ | 0.75±0.04^c^ |
| B | 2-Methoxy-5-methylphenol | 4.46±0.12^c^ | 4.64±0.34^bc^ | 5.41±0.10^a^ | 4.97±0.29^ab^ |
|  | Furfural | 98.58±6.13^d^ | 201.91±14.82^c^ | 254.37±18.98^b^ | 300.25±19.48^a^ |
|  | 5-Methyl furfural | 38.12±1.45^c^ | 49.36±2.95^b^ | 57.37±1.59^a^ | 57.44±2.31^a^ |

^a–b^ Different letters in the same row indicate statistically significant differences at P <0.05 (n=3).

* U0: 1.5%NaCl + UT 600 w; U1: 1.5%NaCl + 0.125%KCl + 0.125%CaCl_2_ + UT 600 w; U2: 1.5%NaCl + 0.25%KCl + 0.25%CaCl_2_ + UT 600 w; U3: 1.5%NaCl + 0.50%KCl + 0.50%CaCl_2_ + UT 600 w.

A: The key volatile flavor compounds (ROAV ≥ 1); B: the potential volatile flavor compounds (0.1 ≤ ROAV ＜ 1)

AU: area units resulting of counting the total ion chromatogram (TIC) for each compound.

**TABLE S3. The ionic strength of four treatment groups (U0, U1, U2 and U3) ***

| Treatments | NaCl  (mol/kg) | KCl  (mol/kg) | CaCl_2_  (mol/kg) | Ionic strength of salts  (mol/kg) |
| --- | --- | --- | --- | --- |
| U0 | 0.407 | 0 | 0 | 0.407 |
| U1 | 0.407 | 0.027 | 0.052 | 0.486 |
| U2 | 0.407 | 0.053 | 0.104 | 0.564 |
| U3 | 0.407 | 0.106 | 0.208 | 0.721 |

***** U0: 1.5%NaCl + UT 600 w; U1: 1.5%NaCl + 0.125%KCl + 0.125%CaCl_2_ + UT 600 w; U2: 1.5%NaCl + 0.25%KCl + 0.25%CaCl_2_ + UT 600 w; U3: 1.5%NaCl + 0.50%KCl + 0.50%CaCl_2_ + UT 600 w.
